# Mathematical Modeling of Human Glioma Growth Based on Brain Topological Structures: Study of Two Clinical Cases

**DOI:** 10.1371/journal.pone.0039616

**Published:** 2012-06-28

**Authors:** Cecilia Suarez, Felipe Maglietti, Mario Colonna, Karina Breitburd, Guillermo Marshall

**Affiliations:** 1 Laboratorio de Sistemas Complejos, Departamento de Computacion, Facultad de Ciencias Exactas y Naturales, Universidad de Buenos Aires, Buenos Aires, Argentina; 2 Servicio de Neurocirugia, Hospital Aleman, Buenos Aires, Argentina; University of Michigan School of Medicine, United States of America

## Abstract

Gliomas are the most common primary brain tumors and yet almost incurable due mainly to their great invasion capability. This represents a challenge to present clinical oncology. Here, we introduce a mathematical model aiming to improve tumor spreading capability definition. The model consists in a time dependent reaction-diffusion equation in a three-dimensional spatial domain that distinguishes between different brain topological structures. The model uses a series of digitized images from brain slices covering the whole human brain. The Talairach atlas included in the model describes brain structures at different levels. Also, the inclusion of the Brodmann areas allows prediction of the brain functions affected during tumor evolution and the estimation of correlated symptoms. The model is solved numerically using patient-specific parametrization and finite differences. Simulations consider an initial state with cellular proliferation alone (benign tumor), and an advanced state when infiltration starts (malign tumor). Survival time is estimated on the basis of tumor size and location. The model is used to predict tumor evolution in two clinical cases. In the first case, predictions show that real infiltrative areas are underestimated by current diagnostic imaging. In the second case, tumor spreading predictions were shown to be more accurate than those derived from previous models in the literature. Our results suggest that the inclusion of differential migration in glioma growth models constitutes another step towards a better prediction of tumor infiltration at the moment of surgical or radiosurgical target definition. Also, the addition of physiological/psychological considerations to classical anatomical models will provide a better and integral understanding of the patient disease at the moment of deciding therapeutic options, taking into account not only survival but also life quality.

## Introduction

Gliomas are a disparate group of primary brain tumors that share the ability of penetrating diffusely throughout the brain, though rarely metastasize outside the central nervous system [1], [2]. The vast majority of gliomas are from the astrocyte (astrocytomas) or the oligodendrocyte (oligodendrogliomas) lineage, or a mixture of both (oligoastrocytomas). Among them, the glioblastoma (grade IV astrocytoma) is, after the non-malignant meningioma, the most frequent type of primary brain tumor and that with worse prognosis [3]. Gliomas usually evolve from lower (neoplasic) to higher (anaplastic) grades with time, possessing four grades (I, II, III and IV) for astrocytomas and two grades (II and III) for oligodendrogliomas and oligoastrocytomas. Most glioblastomas, nevertheless, occur ‘de novo’ (primary glioblastoma) without a previous stage of low grade astrocytoma. Although the ‘apex cell’ (cell of origin) of gliomas is nowadays a subject of research, many support the hypothesis that the majority of gliomas are derived from a ‘glioma stem cell’: the malignization of a neuroglial stem cell or a glial progenitor, rather than the de-differentiation of a mature glial cell [4], [5], [6], [7].

Despite all modern therapies (surgery combined with chemo and radiotherapy), glioblastoma has a life expectancy, after diagnosis, of only around 14 months. This constitutes a real challenge for present day oncology. Molecular genetics revealed that glioblastomas are a group of heterogeneous diseases with common histological features but multiple sets of genetic mutations and epigenetic deregulations that make them have different prognosis and therapeutic responses [8], [9]. This somehow explains why there are reports of patients with glioblastoma outliving up to 10 years and others dying within 2–3 months [10]. In fact, the wide infiltrative area characteristic of gliomas is known to be underestimated by imaging technology of standard use at present: computed tomography (CT) and magnetic resonance imaging (MRI). Surely, this is one of the main reasons of tumor recurrence after surgery.

There has been a great controversy in the last decade about the adequate level of resection in glioma treatment because of issues brought up against the design of earlier studies [11], [12]. Nevertheless new reports seem to agree with the idea that the extent of resection influences the patient’s outcome [13]. In high grade gliomas, gross total tumor resection is associated with longer survival and it is advised to be performed whenever possible [14], though subtotal resections as low as 78% also correspond to a survival advantage [15]. Even older patients can benefit from maximum treatment procedures as they tolerate aggressive surgery without increased surgery-related morbidity [16], [17]. On the other hand, a study that discriminated high grade astrocytomas from high grade oligodendrogliomas found that complete resection significantly increased overall survival only in the former [18]. In relation to low grade gliomas, available data in literature also argue in favor of achieving maximal resection of the tumor for improving survival and delaying tumor progression in hemispheric gliomas, but also in those limited to certain specific subregions [19], [20]. There are even new approaches that propose “supramaximal” or “supratotal” resections of low grade tumors to remove as much tumor cells as possible and delay the anaplastic transformation [21], [22].

Biomathematical models able to accurately predict the evolution of the tumor-mass and infiltrative area may also be of great help when looking for better survival outcomes. Integrative mathematical oncology, a new discipline involved in the development of mathematical models of tumorigenesis, validated by experimental and clinical observations and aimed to reconcile molecular reductionistic with quantitative holistic approaches, has a potential useful role in this type of complex diseases [23]. Models that include tumor evolution in ‘virtual patients’ may result in more realistic predictions helping clinicians to make the best treatment choice, particularly when these models are made patient-specific by using parameter values derived from a given patient. Consequently, mathematical models of glioma evolution in humans have been developed, based on cell proliferation and invasion, and aimed at a better prediction of the real tumor infiltrative area and tumor response after surgery, chemo or radiotherapy [24], [25], [26]. Recently, this class of models include the influence of micro-environmental conditions such as hypoxia, necrosis and angiogenesis [27], [28].

Here, we included different brain topological structures in a patient-specific 3D model of glioma growth based on the proliferation/migration basic design with main coefficients derived from real data. The model allows the spatial correction of the migration coefficient (and consequently the infiltrative area) in correspondence with different brain zones and also the correction of the estimated survival time based on tumor location. These inclusions have the final aim of helping in the determination of potential targets for surgery, radiosurgery and/or stereotaxic radiotherapy. Finally, the inclusion of the Brodmann areas allows the prediction of the brain functions that are being affected as the tumor evolves, offering the possibility of predicting or estimating correlated symptoms. This could be of great value in the estimation of the consequences of whether letting the tumor grow or carrying out neurosurgery. The addition of physiological/psychological considerations to the previous anatomical glioma growth model will surely provide a better and integral understanding of the patient disease at the moment of deciding therapeutic options, taking into account not only survival but also life quality.

## Methods

The mathematical model consists in a reaction-diffusion partial differential equation (PDE) describing the growth and invasiveness of a glioma inside a human brain (partially based on a model presented in Swanson [29]). It may be written as:
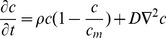
(1)where 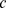 is the concentration of tumor cells in a given node 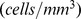, 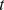 the time 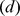, 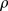 the net proliferation rate of tumor cells 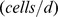, 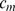 the tumor-cell carrying capacity of the system 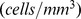 and 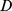 the diffusion rate 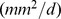 of tumor cells. In the right hand side, the first and second terms represent proliferation and migration, respectively. The proliferation term describes the tumor mass growth by a logistic law that depends on a net proliferation rate (cell proliferation rate minus cell death rate). The migration term describes the tumor infiltration or invasion through peripheral normal brain tissue by the Fick’s diffusion law, assuming that cell migration has a diffusive behavior. This term is dependent on a diffusion rate that is assimilated to a cell migration rate.

The tumor evolves, from an initial unique tumor cell, through a 3D human brain atlas developed by the McConnell Brain Imaging Center from the Neurological Institute of Montreal (www.bic.mni.mcgill.ca/brainweb, [30], [31]). This atlas is based on a simulated brain database (BrainWeb) with a series of digitalized MRI images of human brain slices that cover the whole human 3D brain with a spatial resolution of 1 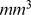. It includes the intracranial distribution of the different component tissues (white and gray matter, connective tissue, vessels, cerebrospinal fluid, dura mater, skull, marrow, muscle, fat and skin). A second atlas discriminating brain structures at different levels (hemispheres, lobes, gyrus, tissues and cell types) was superimposed to it. The Talairach Daemon database (www.talairach.org, [32], [33]) was developed by the Research Imaging Center of the University of Texas Health Science Center San Antonio (UTHSCSA) and it is based on the brain labels present in the Talairach atlas [34]. As it also considers the Brodmann areas (cortical areas that have been related to specific functionalities [35]), it offers the possibility of estimating the main neurological functions that may be affected during tumor evolution. The digitalized Talairach atlas, having a spatial resolution of 5 mm, was adapted to satisfy coordinate-accordance with the Brainweb atlas.

The simulated tumor presents an initial state only with cellular proliferation (benign tumor) and a subsequent advanced state where cellular infiltration starts (malignization of the tumor). This infiltration is structural-dependant, meaning that the net diffusion rate is variable depending on the brain topological structure present in each spatial point of the brain. It is known that glioma cells, like neuroglial stem or glial progenitor cells, migrate mainly along the white matter tracks tending to avoid neuronal accumulations like deep brain nuclei [36]. Indeed, recent data suggest that glioma cell infiltration recreates key aspects of glial progenitor migration [37]. So, after extracting the basal diffusion coefficient from patient’s MRI images, we considered it to be about five times larger for white matter than for gray one, as previously reported [29]. In addition to this, and based on clinical medical expertise of our own and others, we included a 20% larger diffusion coefficient inside the corpus callosum and optical track; and a 20% smaller coefficient inside medulla, brainstem, pons and midbrain, than in the rest of the white matter. For gray matter, we included a 20% smaller diffusion coefficient inside basal nucleus (striatum, globus pallidus, substantia nigra, subthalamic nucleus, lentiform nucleus, amygdala and claustrum), than in cortex. These percentages were chosen in order to fit clinical data.

**Table 1:** Main parametric values used in simulations.

Parameter	Value	Parameter	Value
p1	0.107 *cells*/*d*	p2	0.0107 *cells*/*d*
Dw1	0.255 *mm* ^2^/*d*	Dw2	0.805 *mm* ^2^/*d*
Dg1	0.051 *mm* ^2^/*d*	Dg2	0.161 *mm* ^2^/*d*
*d_diag_*1	18.26 *mm*	*d_diag_*2	16.98 *mm*
*d_let_*	70 *mm*	*C_mass_*	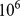 *cel*/*mm* ^3^
*C_inv_*	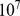 *cel*/*mm* ^3^	*C_max_*	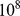 *cel*/*mm* ^3^

p1: net proliferation rate for case 1, p2: idem for case 2, Dw1: net diffusion rate in white matter for case 1, Dw2: idem for case 2, Dg1: net diffusion rate in gray matter for case 1, Dg2: idem for case 2, 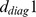: tumor diameter at diagnosis for case 1: 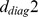: idem for case 2, 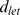: lethal tumor diameter [58], 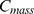: tumor cell concentration at the limit of the tumor mass, 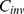: tumor cell concentration that determines invasion (malignization) of the tumor, 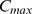: tumor cell carrying capacity [26].

The mathematical model was approximated by finite differences, using standard relaxation techniques, and implemented in MATLAB. Simulated 3D tumor images were generated by the NIFTI package. Although some parameter values were estimated in accordance to previous bibliography (see table 1 for details), the main parameters (net proliferation and migration rates) were derived from the patients under study, thus making the simulation patient-specific. These values were calculated based on tumor-mass and infiltrative areas evidenced, respectively, by T1-gadolinium and T2 MRI brain scans from the patient, as it is generally accepted. Indeed, the peritumoral edema observed in T2 MRI images is partly due to vasogenic mechanisms but also to a microscopic extension of sparse tumor cells [38], so we considered this area as the visible infiltrative area of the tumor. By analyzing two MRI scans of the patient at different times, with no therapeutic intervention between them, two approximations can be applied to estimate 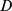 and 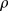. The Fisher’s approximation to calculate the radial velocity of the tumor mass (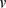) (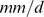) [39], [40]:
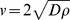
(2)and [41], [25]:
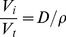
(3)being 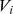 and 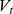 the estimated volumes of the infiltrative area and the tumor mass, respectively. Radius and areas involved were calculated from these images using the ImageJ software, and estimated volumes were derived from them, assuming spherical shapes. The basal diffusion coefficient D derived from equations 2 and 3 was considered to be Dw or Dg depending on tumor initial location (in white or gray matter, respectively).

The relation 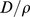 is defined as the invisibility index. As the infiltrative area in gliomas is known to be more extended than what it is observed even by the T2 MRI scan (that it is assumed to loss at least 2% of the real area of tumor invasion [25]), the invisibility index gives an idea of how dangerous the tumor is and what are the eventual consequences of a surgery on it. Indeed, in some cases tumors can be cultured from normal-appearing tissue 4 cm from the edge of glioblastomas [42]. So, the estimation of the tumor growth and invasion parameters in a patient at a certain moment is aimed at the generation of a ‘virtual patient’ able to mimic the evolution of the real disease. Eventually, this may help the clinician to determine the prognosis at the moment of taking complex decisions based on risk/benefit relationships.

The simulation indicates the diagnosis time based on the tumor area detected at the first MRI scan available. As tumor mass evolves, it also calculates the relative extension of each Brodmann area (size of tissue affected relative to the total size of that area) that is being affected. In this kind of prediction of structures or Brodmann areas affected by tumor growth, tumor infiltration is not considered (only tumor mass is taken into account). It has been shown that invasion and spread of low grade tumor cells throughout large areas of the brain happens very slowly over the course of years without interrupting normal regional functionality [1]. The amount of tumor cells in a tissue with detectable altered signal characteristics (shown by an MRI scan) may be as low as 20% and, with this tissue composition, independent functioning is still possible. Due to the great plasticity of the brain, even a large tumor mass invasion with related neuronal damage may not evidence symptoms. Previous data resulting from Parkinson’s disease studies report that most patients have lost 60 to 80 percent or more of the dopamine-producing cells in the substantia nigra by the time symptoms appear [43]. Therefore, here we assumed that there is necessary more than a 60% of a given Brodmann area affected by the tumor mass for related symptoms to become perceptible.

The relationship between Brodmann areas and related symptoms can be extracted from the Brodmann’s Interactive Atlas developed by the Department of Radiology of the Miami Children’s Hospital (www.fmriconsulting.com/brodmann/index.html). The inclusion of the Brodmann areas in the model is aimed to accomplish a prediction of different brain functions that may be affected as tumor evolves, and not to obtain a diagnosis time based on the appearance of this type of symptoms. Indeed, first symptoms that usually leads to a diagnosis are rather nonspecific: fatigue, sleep disturbance, headache and seizures [10]. Only a more detailed analysis of cases whose diagnosis is based on cognitive impairment appearance may be useful for testing this hypothesis.

The survival time was estimated on the basis of tumor size and the site where the tumor is located. In this way, we included in the model a higher death risk as tumor mass invades areas around the foramen magnum. Previous models only accounted for the tumor size, but it is known that 73% of the time, the cause of death in glioma patients is brain herniation (mainly at the place called ‘foramen magnum’, in the base of the skull). Herniation puts extreme pressure on local tissues cutting off the blood supply, inducing comma and eventually the death of the patient. In the rest of the cases, death is finally due to pulmonary embolism, infection, bowel perforation from steroid use or seizures [10].

**Figure 1:**
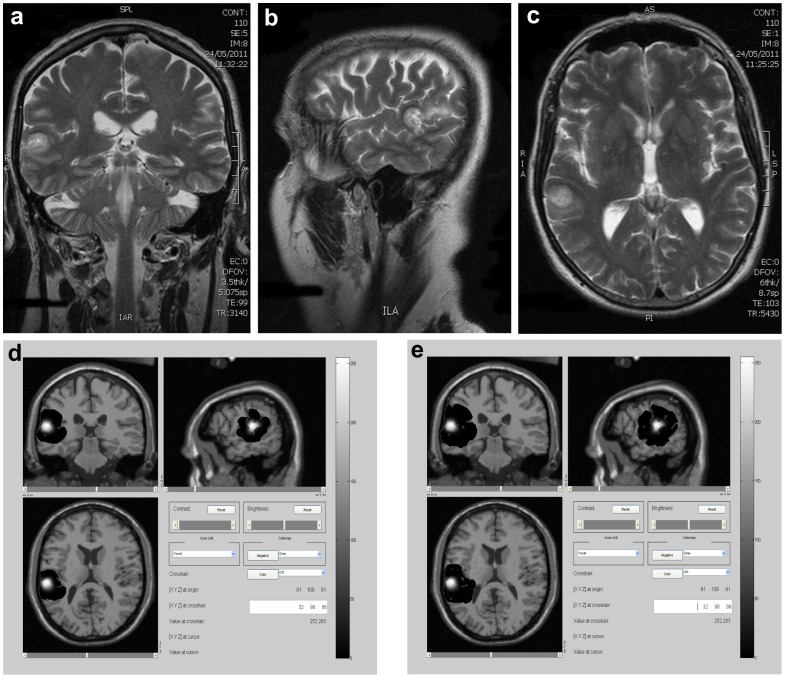
Case 1 at diagnosis. a) Coronal, b) Sagital and c) Axial view of T2 MRI real images of the tumor. d) and e) Corresponding simulated MRI images generated at a detection level of d) 400 cells/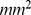 and e) 1 cell/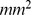. In simulations, white areas represent the tumor mass and black ones, tumor infiltration.

## Results and Discussion

### Simulation of Case 1

Case 1 is a 63 years old male patient, under follow up in our hospital, with a grade IV glioblastoma (determined by histopathology) in the right temporal lobe. As it can be observed in figure 1, at the time of diagnosis the tumor is located very close to the cortex. This corresponds with a higher probability of seizures, symptom that led this patient to a first medical consultation. Patient-specific analysis of this glioma determined that it was characterized by a net proliferation rate of 0.107 cells/day and a net diffusion (migration) rate of 0.255 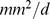 in white matter. Corresponding tumor mass velocity and invisibility index were 120.8 mm/year and 2.38 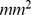, respectively. Considering the range of values reported for high grade gliomas (velocities of 10 to 200 mm/year and invisibility index of 2 to 20 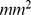 [25]), those values meant that this tumor had a relatively intermediate growth velocity and low invisibility index. These parameters describe a relatively ‘benign’ tumor in relation with the glioblastoma spectrum. In correspondence with (but independently from) this prediction, this was a relatively rare case that was left without surgical intervention up to five months from diagnosis.

**Figure 2:**
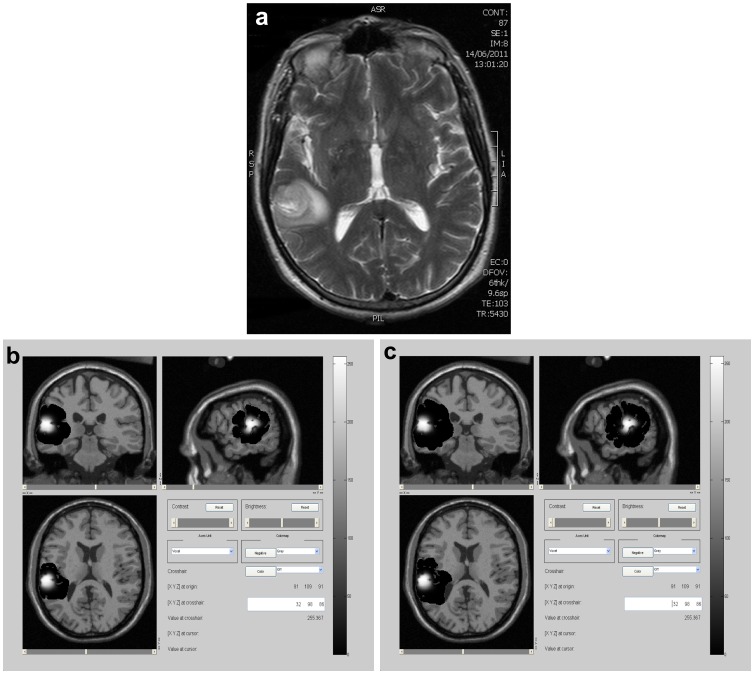
Case 1, 20 days after diagnosis. a) Axial view of a T2 MRI real image of the tumor. b) and c) Simulated MRI images of central slices of the tumor generated at a detection level of b) 400 cells/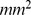 and c) 1 cell/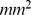. In simulations, white areas represent the tumor mass and black ones, tumor infiltration.

**Figure 3:**
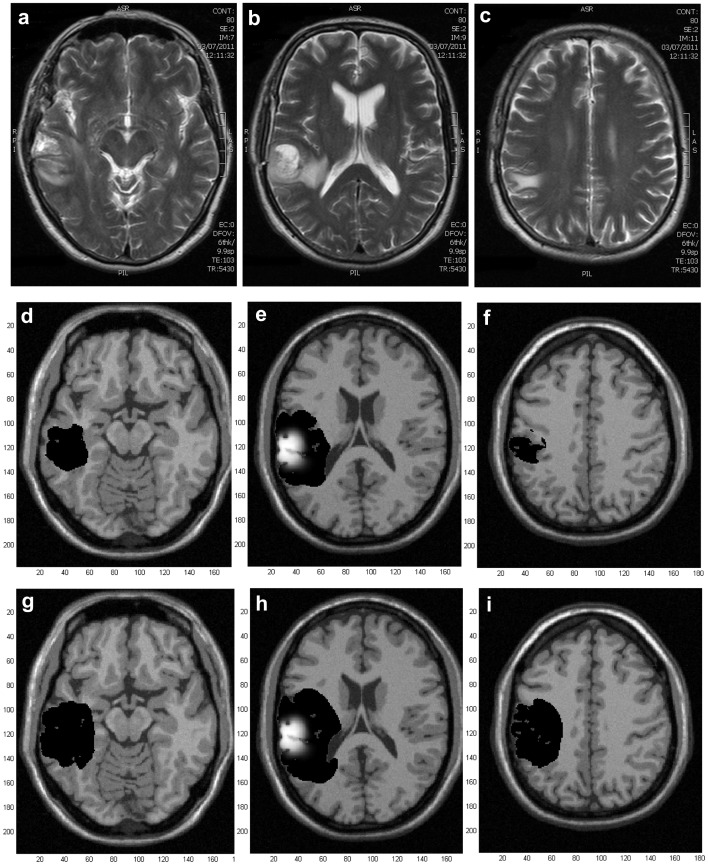
Case 1, 40 days after diagnosis. a), b) and c): Different slices of T2 MRI real images of the tumor in the axial plane. d), e), and f): Corresponding simulated slices generated at a detection level of 400 cells/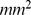. g), h), and i): Corresponding simulated slices generated at a detection level of 1 cell/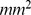. In simulations, white areas represent the tumor mass and black ones, tumor infiltration.

Simulation of case 1 in a ‘virtual patient’ began with the localization of an initial unique tumor cell in the white matter of the superior temporal gyrus. Malignization of the tumor was predicted around 152 days (five months) after its onset and diagnosis after 267 days (nine months). The whole simulation of this case can be observed in the File Case S1 video submitted as a supporting information file. Figure 1 shows the real and simulated tumor at the diagnosis time for different tumor views. In simulated images (figures 1(d) and 1(e)), the central white area represents the concentrated tumor mass while the black surrounding area indicates tumor infiltration, with low concentration of tumor cells invading normal brain tissue.

**Figure 4:**
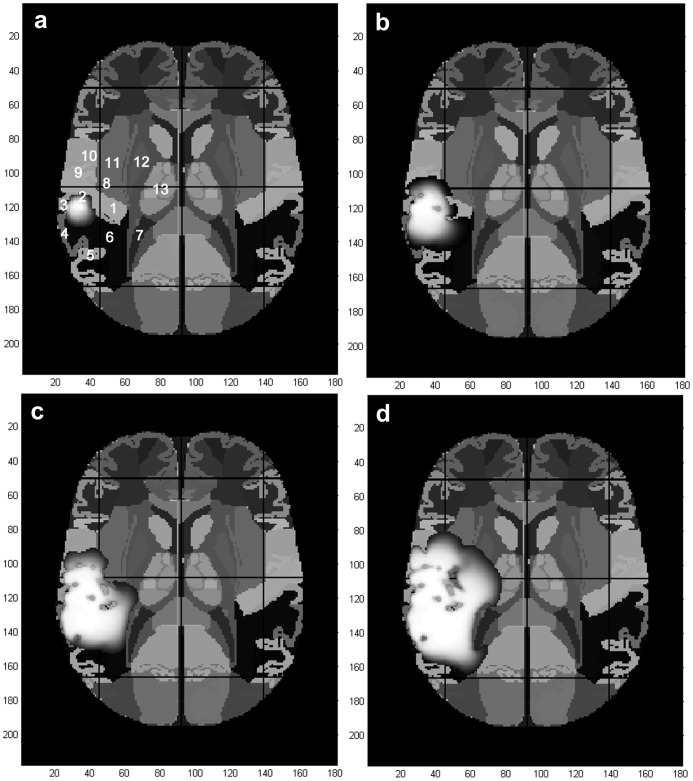
Case 1, predicted Talairach structures affected by the tumor mass during its development. a) At diagnosis, b) 50 days after diagnosis, c) 100 days after diagnosis and d) 140 days after diagnosis (time of death). Tumor detection level at 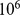 cells/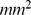. Infiltrative areas have been omitted. Numbers indicate brain structures: 1: transverse gyrus, temporal lobe; 2: Brodmann area 41; 3: Brodmann area 42; 4: Brodmann areas 22; 5: Brodmann area 39; 6: superior gyrus, temporal lobe; 7: lateral ventricle; 8: Brodmann area 13; 9: Brodmann area 43; 10: precentral gyrus, frontal lobe; 11: insula; 12: lentiform nucleus; 13: thalamus.

**Figure 5:**
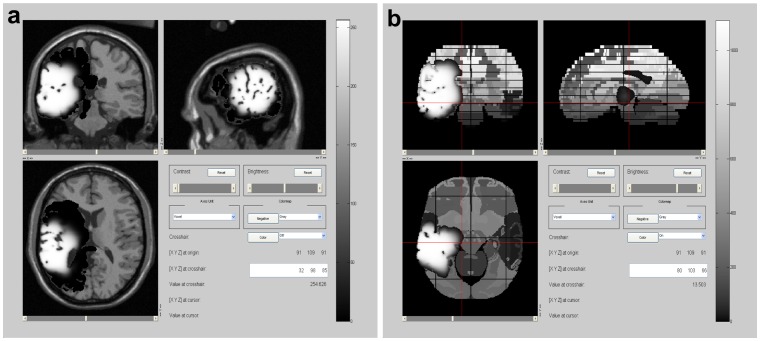
Case 1, predicted tumor evolution at death. a) Simulated MRI images of central slices of the tumor generated at a detection level of 1 cell/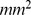. White areas represent the tumor mass and black ones, tumor infiltration. b) Simulated tumor mass near the area of the ‘foramen magnum’ evidenced by the Talairach atlas. Here, infiltrative areas have been omitted.

A good correlation between real and simulated radios of the tumor mass can be observed (8.9 vs. 9.7 mm, respectively). When the simulated image is generated with the same detection level as the MRI technique (around 400 cells/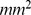 [29]), there is also a fairly good correlation between real and simulated infiltrative areas (compare figure 1(c) with 1(d), axial view). Simulated infiltrative areas detected at 1 cell/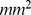, nevertheless, indicate that they would be underestimated by current diagnostic imaging. This is something also reported by others [25] and it is related to one of the main potential clinical utilities of this type of models.

**Figure 6:**
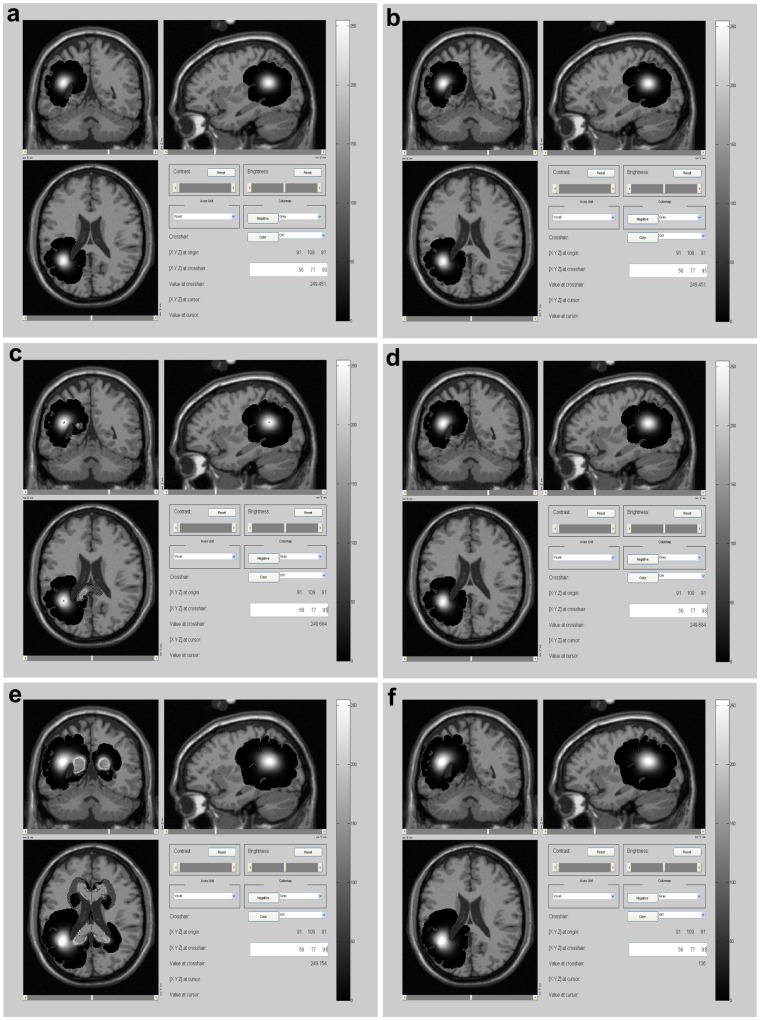
Case 2, comparison between models. Simulated MRI images of central slices of the tumor generated at a detection level of 400 cells/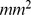. Left: simulations generated by the present model. Right: simulations generated by a previous model from bibliography. a) and b) at diagnosis. c) and d) 18 days after diagnosis e) and f) 100 days after diagnosis.

Figure 2 presents the tumor mass and infiltration 20 days after diagnosis time. At this time, real and simulated tumor mass radios are 12.1 and 12.2 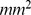, respectively. Simulated infiltrative areas also correlate well with those derived from real images when generated at the same detection level (see figures 1 and 1, axial view), but a higher level of detection indicates again that this area may be larger than that evidenced by real MRI images. These two moments (diagnosis time and 20 days after) were used to estimate the main parameters of the model (net proliferation and diffusion rates) in a patient-specific way. Tumor evolution to the next time stage (40 days after diagnosis) was then predicted by the model.

Figure 3 shows the predicted tumor-mass and infiltrative areas 40 days after diagnosis, over different slices from the axial plane, given a better overview of the 3D tumor evolution. A good correspondence can be observed between real and simulated images generated at a detection level of 400 *cells*/*mm* ^2^ (compare first with second row of images). Here, tumor mass radii are 14.8 and 15.8 mm, respectively. Again, a higher detection level of 1 cell/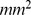 indicates larger invasion than that observed by MRI real images (third row). These results indicate a fairly good prediction accomplished by the numerical model.

Figure 4 superimposes the predicted tumor mass at different times upon the Talairach atlas, indicating the main brain structures that are being affected by tumor evolution. At diagnosis time (270 days from tumor onset, figure 3), tumor mass affects 8.2% of the Brodmann area 41 and 12.6% of the Brodmann area 42 (references 2 and 3, respectively). These areas belong to the primary auditory cortex (Heschl’s gyrus). Related main functions are basic processing of auditory stimuli (speech and non-speech) [44], [45] and processing of sound intensity [46], [47].

50 days after diagnosis (320 days from tumor onset, figure 4(b)), the tumor mass invades great part of transverse and superior gyrus of the temporal lobe (references 1 and 6, respectively) and 10.9% of the Brodmann area 22 (reference 4). If the dominant cerebral hemisphere were the right one, this area would be part of the Wernicke’s area, fully related to auditory comprehension of language [48]. If it was not, as in the majority of cases, this area would have also roles related with receptive language but in a subordinated way, leading to a bilateral dissociation design [49]. This seems to be the case with functions related to lexical ambiguity resolution, where left and right Wernicke’s areas function as processors of dominant and subordinated meanings of ambiguous words, respectively.

100 days after diagnosis (370 days from tumor onset, figure 4(c)), the tumor has expanded to the pre-central gyrus of the frontal lobe (10) and up to the right lateral ventricle (7). It also affected 21.4% of the Brodmann area 43 (9), 10.7% of the Brodmann area 13 (8) and 3.3% of the Brodmann area 39 (5). Brodmann area 43 is associated with some motor responses to vibrotactile digit stimulation [50] and to spoken language [51]. Brodmann area 13 belongs to the insular cortex, that is reported to participate in somatosensory [52], olfaction and taste [53], and verbal memory functions [54], among others. Finally, Brodmann area 39 is also part of the Wernicke’s area or its contralateral homologous. In the right hemisphere, it is generally associated to visuospatial processing [55] and music reading [56]. Figure 4(d) predicts that, at the time of death (survival time of 140 days after diagnosis or 410 days from tumor onset), the tumor mass has extended through the insula (11), lentiform nucleus (12) and thalamus (13).

Figure 5 presents tumor status at the predicted time of death. The estimated tumor mass diameter (assuming an spherical shape) at this moment is 62.9 mm, a value slightly smaller than the lethal size parameter used (70 mm, see table 1). This might be attributed to the corrections added to the model in relation with tumor location. Indeed, at this time the tumor mass edge reaches the ‘foramen magnum’, as is evidenced by the Talairach atlas in figure 5(b). This means that herniation, and consequently death, could be imminent.

### Simulation of Case 2

Case 2 is a 32 years old male with a histopathology of anaplastic oligodendroglioma (grade III) reported in previous literature [57]. The whole simulation of this case can be observed in the File Case S2 video submitted as a supporting information file. Figure 6 compares simulations of this case at different times made with two distinct mathematical models: left column corresponds to simulations obtained with the present model, right column corresponds to simulations derived from our implementation of a model previously described in the literature [29] (this model does not include differential migration based on topological brain structures). This figure should be compared with figure 1a and 1c from [57] that shows the real MRI images from this patient. Real tumor dimensions correspond well with those predicted by the present model. At diagnosis, real and simulated tumor mass radii are 8.9 and 8.7 mm; while tumor infiltration radii are 28.4 and 28.9 mm, respectively (compare figure 1a from [57] with axial view from figure 6(a)). Eighteen days after diagnosis, real and simulated tumor mass radii are 11.4 and 10.6 mm; and tumor infiltration radii are 33.1 and 31.2 mm, respectively (compare figure 1c from [57] with axial view from figure 6(c)). Simulations made with a previous model (figures 6(b) and 6(d)) also reach tumor dimensions similar to real ones.

However, if we analyze tumor spreading the situation is different. Our model seems to be more accurate regarding tumor spreading than previous ones and these differences between models become significant 18 days after diagnosis. Real images show that tumor invasion through the corpus callosum is significantly augmented at that time (figures 1c and 1d from [57]). They also evidence a mass-effect that compresses the lateral ventricles. Although this kind of mathematical models do not include the mass-effect, this type of spreading through the corpus callosum can be observed in simulations made with our model (see axial view form figure 6(c)) but not in those derived from previous ones (axial view from figure 6(d)).

This consideration is extremely important, as its consequences worsen with time: differences between both models become more evident 100 days after diagnosis, as it can be evidenced in figures 6(e) and 6(f). At this time point, simulations made with the present model predicts a larger spreading of the tumor through the corpus callosum with tumor cells reaching the contralateral hemisphere and anterior zones of the brain. Simulations with previous models, on the contrary, do not evidence this type of spreading. Though this time stage does not have real images to correlate with, it evidences the natural evolution of the previous tumor status.

### Conclusions

Despite all modern therapies, gliomas continue to be a challenge for present oncology, mainly due to their great invasion capability. This makes surgical or radiosurgical ‘security margins’ often miscalculated, leaving in the tissue viable tumor cells that eventually are the cause of treatment failure. The development of more realistic mathematical models able to accurately predict the extent and distribution of tumor cell infiltration through normal brain tissue would be an additional predictive tool of clinical utility.

In this paper we aimed to make a contribution to present models of glioma growth in humans through the introduction of brain topological structures into a 3D patient-specific mathematical model based on cell proliferation and invasion through normal tissue. This allows the correction of cell infiltration margins, life expectancy and, with the inclusion of Brodmann areas, the eventual prediction of brain functions that could be affected by the tumor mass or by an eventual therapeutic intervention.

The model was applied to two clinical cases, making accurate predictions based on parameters obtained from MRI images of the patients involved. Patient-specificity is a very important characteristic of present models of glioma growth, as parameter values extracted from average population are not valid due to the great variety of glioma subclasses. The analysis of tumor spreading predictions made by our present model shows, in the second clinical case, that it is able to accomplish a better prediction of the real tumor state than that achieved by previous models present in literature.

This type of models could guide the surgeon to better decisions based on information about how the tumor will evolve in terms of tumor mass, infiltrative areas and their relative relevance for survival or neurological functions. For instance, in one case a more extensive resection would be justified if the tumor has infiltrated an area not compromised with vital functions or not essential in terms of life quality. In another case, on the contrary, it would not worth a larger resection than the tumor mass due to the great extension of the infiltration and/or the relevance of the brain areas involved. Finally, a case with a tumor mass extending to vital structures would justify a more aggressive intervention before it reaches them. Although we checked our model with two high grade gliomas, it can also be applied to low grade ones, as they are also already infiltrating at first diagnosis. The inclusion of differential migration based on topological brain structures in glioma growth is another step towards a better prediction of the extension and shape of tumor infiltration at the moment of surgical or radiosurgical target definition.

## Supporting Information

File Case S1
**Supplemental video of case 1 tumor growth simulation (glioblastoma).** Simulation was run for 500 days since tumor onset (one original cell) with a detection level of 1 cell/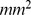.(WMV)

File Case S2 **Supplemental video of case 2 tumor growth simulation (anaplastic oligodendroglioma).** Simulation was run for 2600 days since tumor onset (one original cell) with a detection level of 1 cell/*mm* ^2^.
(WMV)
